# Assessment of risk factors associated with prevalence of gastrointestinal parasites in poultry of central plain zone of Punjab, India

**DOI:** 10.14202/vetworld.2021.972-977

**Published:** 2021-04-22

**Authors:** Malkeet Singh, Paramjit Kaur, Lachhman Das Singla, Neeraj Kashyap, Mandeep Singh Bal

**Affiliations:** 1 Department of Veterinary Parasitology, College of Veterinary Science, Guru Angad Dev Veterinary and Animal Sciences University, Ludhiana; Punjab, India; 2 Department of Animal Breeding and Genetics, College of Veterinary Science, Guru Angad Dev Veterinary and Animal Sciences University, Ludhiana, Punjab, India; 3 Animal Diseases Research Centre, Guru Angad Dev Veterinary and Animal Sciences University, Ludhiana, Punjab, India

**Keywords:** central plain zone, gastrointestinal parasites, India, prevalence, poultry, Punjab, risk factors

## Abstract

**Background and Aim:**

Parasitic diseases are an important hurdle to the economy for the developing poultry industry due to their deleterious effects resulting into malnutrition, diminished feed conversion ratio, weight loss, decreased egg production, and mortality in young birds. The aim of this study was to assess the prevalence and associated risk factors of gastrointestinal (GIT) parasites in poultry farms of central plain zone of Punjab.

**Materials and Methods:**

A total of 490 pooled droppings and 351 intact intestines of poultry from slaughterhouses from seven districts of central plain zone of Punjab state, India, were collected and analyzed from September 2016 to May 2018 by qualitative and quantitative techniques.

**Results:**

An overall prevalence of GIT parasites was 38.36% with significantly (p<0.01) highest (74.1%) in Ludhiana and lowest (12.0%) in Shri Fatehgarh Sahib. The most predominant (86.2%) infection was coccidia. The birds reared under a deep litter system were having a higher (p<0.01) fecal load of helminthic eggs and coccidian oocysts (54.4%) compared to the cage system (37.5%). Infection rate was apparently more (40%) in broilers than layers (35.7%). Prevalence of GIT parasites was higher (p<0.01) in monsoon season (58.5%) and lower in summer season (24.48%). The broilers in the age group of 0-2 weeks possessed a significant higher (p<0.05) level of GIT parasitic infection (57.5%), while in case of layers, a higher infection rate (46.66%) was observed in birds between 9 and 18 weeks of age as compared in other groups. Higher (p<0.05) infection rate of GIT parasites was seen in crossbred (45.55%) birds as compared to desi birds (20.00%).

**Conclusion:**

The study showed that coccidiosis was the predominant infection among all GIT parasites based on fecal and intestinal tract content analysis. The risk factors associated with the prevalence of GIT parasitic infections were geographical location, deep litter system, broilers, age, crossbred breeds, and monsoon season.

## Introduction

Poultry production is increasing rapidly due to the low establishment cost and efficiency of poultry to convert nutrients present in food stuffs into high value animal protein that contributes 30% of the world’s animal protein being consumed by humans. Although the impact of parasitic diseases in birds reared under commercial production systems or cage systems, is decreasing due to modernization in poultry farming and biosecurity measures, the farm birds ­maintained in deep litter system and backyard free-ranging system remains more prone to parasitic infections through consumption of contaminated feed and water by litter droppings and scavenging habits [1]. Thus, gastrointestinal (GIT) parasitism in poultry results in adverse economic effects on production parameters in backyard or farmyard flocks in comparison to confinements rearing. The most prevalent endoparasitic infections in poultry are caused by cestodes, nematodes, and coccidiosis due to *Eimeria* spp. and oviduct fluke infection results in pathogenicity leading to morbidity, mortality, and economic losses [1]. Chronic helminthic infections result in poor growth, reduced egg production, and fertility, while acute infestations result in mortality [2] and only intestinal roundworms have been estimated to cause production losses in the range of 10-20% [3].

In spite of advances made in prevention and control through immunoprophylaxis, chemotherapy, management, and nutrition, coccidiosis remains one of the major problems of poultry [4]. Globally, economic losses incurred due to coccidiosis are estimated to be more than $800 million per annum and in India Rs. 1.14 billion [5]. The clinical form of the disease manifests through prominent signs of mortality, morbidity, diarrhea or bloody feces, and subclinical coccidiosis manifests mainly by poor weight gain and reduced efficiency of feed conversion [6]. Several published reports on the prevalence of the GIT parasites from various states of the country are available from North Indian region [7], Jammu [8], South Gujarat [9], Madhya Pradesh [10], and Bangalore [11].

The scrutiny of the literature on the prevalence of GIT parasites in relation to associated risk factor in poultry from different agroclimatic zones of Punjab is lacking. Hence, the aim of the present investigation was to assess the prevalence and associated risk factors with intestinal parasitism in poultry from the central plain zone of Punjab, India.

## Materials and Methods

### Ethical approval

The study was approved by the Institutional Animal Ethics Committee of Guru Angad Dev Veterinary and Animal Sciences University (IAEC/2016/532-539).

### Study area and period

Punjab state extends from the latitudes 29°30’ N to 32°32’ N and longitudes 73°55’ E to 76°50’ E. It covers a geographical area of 50,362 km^2^, which is 1.54% of country’s total area and lie between altitudes 180 m and 300 m above mean sea level. Average rainfall in Punjab is 565.9 mm and ranges from about 915 mm in north to 102 mm in the south. Among the five agro-climatic zones of Punjab, central plain zone (study area) is the largest constitutes seven districts, namely, Amritsar, Tarn Taran, Kapurthala, Jalandhar, Ludhiana, Shri Fatehgarh Sahib, and Patiala. The study was conducted from September 2016 to May 2018.

### Sample collection

Pooled fecal droppings (n=490) were collected from crossbred (Punjab 1), commercial (Sugna, Galaxy, and Vencobb), and desi breeds of poultry in properly labeled sterile polythene bags, kept in a cool transport box, and were brought to the laboratory for examination. Proper history and other details were noted in the questionnaire so as to look various risk factors, namely, age, season, type of management, and treatment given. Intact intestines (n=351) were randomly collected from naturally dead birds of different age groups from slaughtered houses and local meat shops of the above-mentioned districts of central plain zone of Punjab.

### Fecal sample analysis

Fecal samples were subjected to detailed parasitological analysis for the presence of parasitic eggs/oocysts by standard sedimentation and flotation techniques [2]. The samples found positive for parasitic eggs/oocysts were subjected to quantitative technique (McMaster egg counting technique) to get the eggs per gram and oocysts per gram (OPG) [12].

The intestines collected were dissected for the presence of parasites and observed for any pathological lesions. Intestines were tied at different portions (upper, middle, and lower part of small intestine, large intestine, and ceca) before cutting. Wet smears were prepared from the mucosa of respective gut portions and examined under the microscope for the presence of oocysts [2]. The gut samples found positive for coccidian oocysts were further sporulated for *Eimeria* species identification. OPG of gut samples was done to estimate oocyst load.

### Risk factors and statistical analysis

Statistical data analysis was performed using Statistical Analysis System for Windows, Version 9.4 (SAS^®^, USA). The effect of risk factors on the prevalence of GIT parasites was determined on the basis of: (a) Management type (deep litter system, cage system, and backyard confined system); (b) bird type (broilers and layers); (c) season (monsoon, winter, and summer); (d) age group in broilers: 0-2, 3-4, 5-6, and 7-8 weeks); layers (0-8, 9-18, 19, and above weeks); and (e) breed (pure, commercial, desi, and cross) using Chi-square test. Variables with the significant association at p<0.05 (two-sided) were subjected to the multivariate logistic regression model. The results were expressed as p-value and odds ratio with a 95% confidence interval.

## Results

### Prevalence

**Figure-1:**
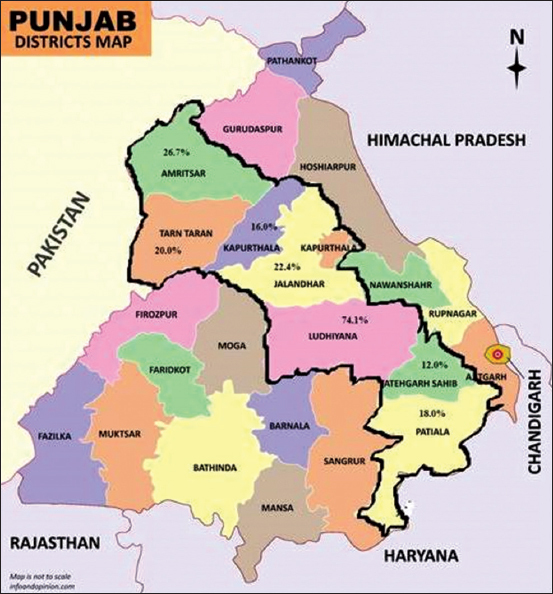
District wise prevalence of gastrointestinal parasites in poultry birds from central plain zone of Punjab (inside black line). Source: https://www.infoandopinion.com/punjab-map-download-free-map-in-pdf/1145

Out of 490 droppings of poultry birds examined from seven districts of central plain zone of Punjab state, 188 (38.36%) samples were found positive for GIT parasitic infections. The occurrence of GIT parasites varied significantly (p<0.01) among the districts and was highest in Ludhiana district (74.1%), followed by Amritsar (26.7%), Jalandhar (22.35%), Tarn Taran (20.0%), Patiala (18.0%), Kapurthala (16.0%), and lowest in Shri Fatehgarh Sahib (12.0%) (Table-1 and Figure-1). The composition of different GIT parasites showed coccidian oocysts to be predominant (86.2%), followed by *Ascaridia galli* eggs (11.2%) and mixed infection of *A. galli* eggs and coccidian oocysts (2.6%).

**Table 1:** Prevalence of gastrointestinal parasites in poultry of seven districts in central plain zone of Punjab.

District	Samples examined	Positive (%)	Ascarid eggs (%)	Coccidian oocysts (%)	Mixed infections *Ascaridia* and coccidia (%)	Oocysts per gram range
Ludhiana	170	126 (74.1)	21 (16.7)	100 (79.4)	5 (4.0)	2200-300,000
Jalandhar	85	19 (22.4)	-	19 (100)	-	2600-12,000
Patiala	50	9 (18.0)	-	9 (100)	-	3200-11,500
Tarn Taran	40	8 (20.0)	-	8 (100)	-	1500-5600
Amritsar	45	12 (26. 7)	-	12 (100)	-	3600-16,800
Kapurthala	50	8 (16.0)	-	8 (100)	-	1600-7600
Fatehgarh Sahib	50	6 (12.0)	-	6 (100)	-	1800-9600
Total	490	188 (38.4)	21 (11.2)	162 (86.2)	5 (2.6)	1500-285,000
χ^2^		143.46*				

* Indicates values varying significantly at 1% level of significance

**Figures-2:**
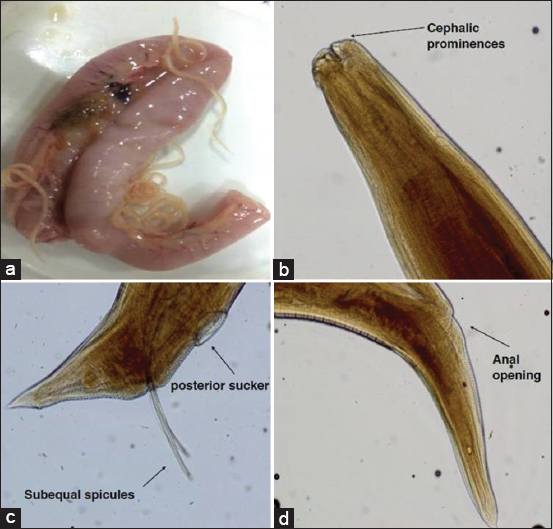
(a) Intestinal blockage due to *Ascaridia galli*. (b) Photomicrograph of anterior end of *A. galli* showing cephalic prominences. (c) Photomicrograph of posterior end of male of *A. galli* showing sub-equal spicules and posterior sucker. (d) Photomicrograph of posterior end of female of *A. galli* showing anal opening and transverse striations.

**Figure-3:**
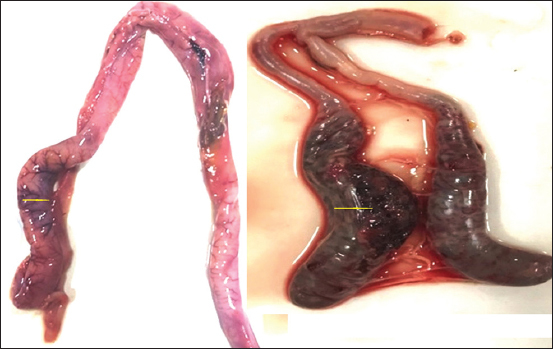
Duodenum and cecal part of intestines affected with coccidian infection.

Out of 351 intestinal tracts examined, gross changes were observed in 83 (23.64%) of the intestinal tracts, revealing 3.6% (3/83) of *A. galli* and 96.4% (80/83) of coccidial infection (Table-2). Similar to the pattern of copro-prevalence, district wise significantly higher infection of GIT parasites was seen in Ludhiana (35.6%) and lowest in district Kapurthala (9.0%). Gross examination of the three GIT tracts revealed nematodes *A. galli* causing blockage of intestine (Figure-2a). Adult worms of *A. galli* were up to 12 cm in length having yellowish-white color and were semitransparent. Cuticle was distinctly striated and the cuticular alae were feebly developed. The photomicrograph of the temporarily mounted parasites in glycerol after clearing in the lactophenol identified based on the character of anterior part of *A. galli* with cephalic prominences (Figure-2b), male possesses sub-equal spicules and posterior sucker (Figure-2c), while female posterior end having transverse striation with anal aperture (Figure-2d). The gross examination of intestinal tracts (80) showed thickening of intestinal mucosa and mucous enteritis. Out of 80 infected intestines, 39 intestines were grossly positive for lesions of intestinal coccidiosis and 41 for cecal coccidiosis (Figure-3). Extreme ballooning and petechial hemorrhages were observed in intestinal form. When opened, discrete hemorrhagic spots were observed on the mucous membrane of the intestine and intestinal contents were yellowish and blood-tinged.

**Table 2:** Prevalence of gastrointestinal parasites based on intestinal tract of poultry of seven districts in central plain zone of Punjab.

Districts	Intestines examined	Intestine positive (%)	*Ascaridia galli* (%)	Coccidia (%)
Ludhiana	104	37 (35.6)	3 (8.1)	34 (91.9)
Jalandhar	52	14 (29.9)	-	14 (100.0)
Patiala	20	2 (10.0)	-	2 (100.0)
Tarn Taran	55	9 (16.4)	-	9 (100.0)
Amritsar	50	13 (26.0)	-	13 (100.0)
Kapurthala	55	5 (09.0)	-	5 (100.0)
Fatehgarh sahib	15	3 (20.0)	-	3 (100.0)
Total	351	83 (23.64)	03(3.6)	80 (96.4)
x^2^	18.904*			

* Indicates values varying significantly at 1% level of significance

### Risk factors analysis

Birds reared on the deep litter system harbor significantly (p<0.05) higher infection (54.4%) than the cage system (37.5%) (Table-3). No bird was found positive from the confined backyard poultry system. Broilers apparently harbored more parasitic infection (40.0%) than layers (35.7%). Season-wise, significantly (p<0.01) highest prevalence was in monsoon season (58.5%) and lowest in summer season (29.4%). A similar pattern of influence of season among broilers was observed.

**Table 3:** Risk factors associated with prevalence of GIT parasites in poultry of central plain zone of Punjab.

Risk factor	Parameter	Total sample examined		Total positive (%)
Management type	Deep litter	90		49 (54.4)
	Cage system	370		139 (37.5)
	Confined backyard	30		0 (0.0)
	Total	490		188 (38.4)
	χ^2^		28.61**	
Bird type	Broilers	300		120 (40.0)
	Layers	190		68 (35. 8)
	Total	490		188 (38.4)
	χ^2^		0.872^NS^	
Season	Monsoon (July-October)	130		76 (58.5)
	Winters (November-February)	125		43 (34.4)
	Summer (March-June)	235		69 (29.4)
	χ^2^		31.0904**	
Age group (broilers)	0-2 weeks	40		23 (57.5)
	3-4 weeks	130		49 (37.7)
	5-6 weeks	65		30 (46.1)
	7-8 weeks	65		18 (27.7)
	χ^2^		10.521*	
Age group (layers)	0-8 weeks	25		06 (24.0)
	9-18 weeks	30		14 (46.7)
	19 weeks and above	135		48 (35.5)
	χ^2^		3.060^ NS^	
Breed	Pure	110		32 (29.1)
	Commercial	245		106 (43.3)
	Desi	45		09 (20.0)
	Cross	90		41 (45.5)
	χ^2^		18.475*	

*, **Indicate values varying significantly at 5% and 1% level of significance

Among broilers, birds of 0-2 weeks age group harbor significant (p<0.05) higher level of infection (57.5%), while, lowest infection was seen in birds of age group of 7-8 weeks (27.69%). Among layers, the high prevalence was observed in birds of age group 9-18 weeks (46.66%) as compared to birds of age more than 19 weeks (35.55%) and 0-8 weeks (24.8%). The copro-prevalence showed the highest (p<0.01) level of infection in crossbred (45.55%), followed by commercial (43.26%), pure (23.09%), and desi (20%) birds.

## Discussion

A significant deviation in prevalence of the GIT parasite in poultry of seven districts of central plain zone of the state may be due to variation in management practices adopted at different farms. The published reports on prevalence of GIT parasites of poultry with associated risk factors from different parts of the world showed great variation (16-80%) [13-16]. The predominance of coccidial infection followed by *A*. *galli* infection in the present findings was similar to earlier reports, from India [17], Cameroon [18], South Gujarat, India [9], and Nigeria [19-21].

The presence of *A. galli* as the only prominent helminthic infection in the present study is in agreement with findings of Puttalakshmamma *et al*. [11]. Besides this, the most important infection of coccidiosis was detected by both on copromicroscopic and intact intestinal tract examination. The higher infection rate of coccidiosis may be due to the fact that majority of the encountered birds were reared on deep litter system with minimal use of anti-coccidial treatment by the farmers. In general, the prevalence of coccidiosis is primarily influenced by management rather than flock size [22,23]. The poor management mainly includes the moist litter that promotes oocysts sporulation, high stocking density, and inadequate ventilation [24]. The birds reared on the deep litter have direct access to the infective stages of ova/oocysts of parasites, so the role of hygienic management plays a significant impact to increase productivity of poultry.

Among different types of birds, broilers harbored more infection. It may be due to the fact that broilers were reared under deep litter system, while layers were mainly from the cage system. Likewise, Agishi *et al*. [25] reported that birds reared on the deep litter system showed significantly higher susceptibility to coccidiosis than the battery system.

Finding of significantly higher GIT parasitic infections in monsoon season is in commensuration of Salam *et al*. [26]. The optimum temperature and relative humidity for development and hatching of eggs or oocysts are 26-29°C and >80%, respectively. There is a strong correlation between the occurrence of coccidiosis, relative humidity, temperature, and rainfall. A significant relationship between the season and prevalence of GIT parasites, particularly coccidial infection in rainy season, has been reported in published data of Zimbabwe [27], Bangladesh [28], and Andhra Pradesh, India [29].

Many researchers reported that all ages of birds are susceptible to coccidiosis, but younger birds are more susceptible to infection than older birds as in the present study, birds of 0-2 weeks age group harbor significantly higher level of infection [25]. In contrast to the present study, more prevalence in adult birds is also reported [30,31].

Various breeds are reared in different states of the country that showed susceptibility to GIT parasites as Banjara breed birds in Odisha [32], local and exotic breeds of chickens in Madhya Pradesh [10], local and exotic breeds of chickens in Bangalore [11], and desi fowl (*Gallus gallus domesticus*) in Andhra Pradesh [29].

## Conclusion

An overall prevalence of GIT parasites based on eggs and oocysts in fecal samples and parasites in intact intestine in chicken of central plain zone of Punjab, indicated coccidiosis as predominant infection. Geographical location, deep litter system, broilers, age, crossbred breeds, and monsoon season were the most important risk factors for the prevalence of GIT parasitic infections. To access the associated risk factors with occurrence of GIT parasites in remaining zones of the state, a comprehensive study is required so that area-based control strategies can be formulated and advised to the poultry farmers.

## Authors’ Contributions

MS: Sample collection, research work in the laboratory, and compilation of the data. PK conceived and designed the research work and did the data analysis. PK and LDS wrote and evaluated the manuscript. NK guided in statistical analysis. MSB helped in sample collection. All authors read and approved the final manuscript.
